# Letter to the Editor Regarding the Article “Evaluating the Predictive Value of the Endothelial Activation and Stress Index in Determining the Severity and Prognosis of Acute Pulmonary Embolism”

**DOI:** 10.1159/mpp/adpag022

**Published:** 2026-09-11

**Authors:** Ramazan Amanvermez, Fatih Çalışkan

**Affiliations:** a Department of Medical Biochemistry, Faculty of Medicine, Ondokuz Mayıs University, Samsun, Türkiye; b Department of Emergency Medicine, Faculty of Medicine, Ondokuz Mayıs University, Samsun, Türkiye

**Keywords:** Acute pulmonary embolism, Endothelial activation and stress index, Prognosis, Biomarkers

Dear Editor,

We have carefully read the recent report by Karayiğit et al. [1] entitled “Evaluating the Predictive Value of the Endothelial Activation and Stress Index in Determining the Severity and Prognosis of Acute Pulmonary Embolism.” The authors contribute to our knowledge on endothelial activation and stress index (EASIX) as a marker of endothelial injury and dysfunction that independently predicts high-risk acute pulmonary embolism (APE) and in-hospital mortality. However, the study presents vascular endothelial dysfunction along with various laboratory parameters in an unsatisfactory way in APE patients, which is a life-threatening cardiovascular emergency associated with high morbidity and mortality. Although EASIX is calculated from routinely available laboratory parameters, its real clinical value lies in the emergency department where rapid risk stratification is essential. Also, they did not discuss how EASIX could be incorporated into the initial diagnostic workflow. Upon hospital admission, it is noteworthy that increased EASIX value is associated with biomarker changes indicative of endothelial and microvascular dysfunction: Decreased platelet count and hemoglobin suggest endothelial-mediated consumptive coagulopathy and microangiopathic haemolysis; abnormal coagulation tests (INR, PT, aPTT) further display coagulation system activation due to endothelial dysfunction, while elevated hepatic enzymes (ALT, AST), bilirubin, lactate, lactate dehydrogenase (LDH), urea, and creatinine think concomitant hepatic and renal damage along with tissue hypoperfusion following endothelial injury [2]. It is assumed that routine test values of the EASIX formula (LDH, serum creatinine, platelet count) may change depending on the severity of APE pathophysiology, underlying risk factors for APE, and comorbidities: LDH activity increases under hypoxic conditions and haemolysis; elevated creatinine represents impaired renal perfusion and systemic vascular injury associated with endothelial dysfunction; and a declined platelet count is a characteristic feature of vascular-associated coagulopathy and endothelial consumption. For instance, they estimated that the platelet count was significantly lower in the intermediate-risk group than in the high-risk group for APE (as shown in Table 1). However, in another study [3], Creatinine was measured lower in the high EASIX score (1.11) group when compared to the training cohort (EASIX score: 1.02) group at first presentation to heart failure outpatient clinic in patients with chronic heart failure. Scoring systems or clinical scoring tools are likely scores developed to help a specialist physician to exclude or confirm pulmonary embolism. In recent years, new biomarkers have been established as vascular endothelial injury markers (sP-selectin, soluble thrombomodulin, vWF, etc.), inflammatory markers (hsCRP, neutrophil-to-lymphocyte ratio, etc.), cardiac markers, and microRNAs in relation to the early diagnosis, severity, and prognosis of pulmonary embolism [4, 5]. Thus, further prospective studies are needed to examine biomarkers as the aforementioned vascular endothelial damage markers and inflammatory markers as well as EASIX in determining the early diagnosis, severity, and prognosis of APE.

## Conflict of Interest Statement

The authors declare that there are no conflicts of interest and any financial support.

## Funding Sources

No funding was received for letter article.

## Author Contributions

The authors equally contributed to conception and design of this article, read, and approved the final version.
